# Causal Links Between Corneal Biomechanics and Myopia: Evidence from Bidirectional Mendelian Randomization in the UK Biobank

**DOI:** 10.3390/bioengineering12040412

**Published:** 2025-04-13

**Authors:** Xuefei Li, Shenglong Luo, Kuangching Lin, Hera Soha, Meixiao Shen, Fan Lu, Junjie Wang

**Affiliations:** 1 National Engineering Research Center of Ophthalmology and Optometry, Eye Hospital, Wenzhou Medical University, Wenzhou 325027, China; lixuefei_0310@163.com (X.L.); luoshenglong1998@163.com (S.L.); lgqd33@163.com (K.L.); herasoha97@hotmail.com (H.S.); smx77@mail.eye.ac.cn (M.S.); 2 National Clinical Research Center for Ocular Diseases, Eye Hospital, Wenzhou Medical University, Wenzhou 325027, China; 3 NMPA Key Laboratory for Clinical Research and Evaluation of Medical Devices and Drug for Ophthalmic Diseases, Eye Hospital, Wenzhou Medical University, Wenzhou 325027, China

**Keywords:** myopia, corneal biomechanics, Mendelian randomization

## Abstract

Background: Myopia is a leading cause of visual impairment worldwide, and accumulating evidence suggests that biomechanics may be closely linked to its development. Understanding this relationship may help clarify the underlying mechanisms of myopia and guide treatment strategies. The aim of the study is to investigate the causal relationship between myopia and corneal biomechanics using the UK Biobank (UKB) database. Methods: Data from 11,064 eyes in the UKB, including refraction results and Ocular Response Analyzer (ORA) measurements, were analyzed. Eyes were categorized by spherical equivalent (SE) into emmetropia, mild myopia, moderate myopia, and high myopia. One-way ANOVA assessed differences in corneal biomechanical parameters across the varying myopia groups, while Quantile Regression (QR) explored the relationship between these parameters and myopia severity across the different quantiles. A Mendelian randomization (MR) analysis was employed to explore the causal relationships. Results: Significant differences in corneal biomechanical parameters and intraocular pressure (IOP) were observed across the myopia levels (*p* < 0.001). High myopia was associated with lower corneal hysteresis (CH), a lower corneal resistance factor (CRF), and increased IOP. The QR analysis demonstrated that lower corneal biomechanics were associated with higher degrees of myopia, with the impact of corneal biomechanics becoming more pronounced as the myopia severity increased. The MR analysis indicated that low CH (OR = 0.9943, *p* = 0.004) and CRF (OR = 0.9946, *p* = 0.002) values were risk factors for myopia, while no causal effect was found when the myopia was treated as the exposure and corneal biomechanics as the outcome. Conclusions: This study establishes a causal relationship where reduced corneal biomechanics contribute to myopia, while myopia itself does not directly affect biomechanics. Corneal biomechanics could serve as a biomarker for assessing high myopia risk. These findings offer new insights into high myopia’s pathological mechanisms and targeted prevention.

## 1. Introduction

Myopia is a growing public health concern worldwide, and its prevalence has reached alarming levels [1]. In some regions, up to 90% of young adults are affected, making it one of the most common ocular disorders [2]. The rising prevalence of myopia has significant public health implications due to its association with severe complications, collectively known as pathological myopia, such as posterior staphyloma, retinal detachment, and myopic maculopathy, which can lead to irreversible vision loss [3].

Due to the severity of pathological myopia, it is crucial to explore the mechanisms and risk factors of myopia progression. Previous studies have attributed this to the stimulation of light signals, which triggers a cascade of biochemical reactions in the retina, choroid, and sclera, leading to scleral fibroblast apoptosis and extracellular matrix remodeling. In highly myopic eyes, the scleral collagen fiber diameters decrease and the spacing becomes narrow [4,5], causing scleral thinning, axial elongation, and pathological myopia [6]. In this process, the scleral biomechanics are of paramount importance, as they dictate the sclera’s actual deformation response to biochemical signaling stimuli [7]. Currently, in clinical settings, air-puff methods are used to induce corneal deformation for measuring corneal biomechanics, while in vivo measurement devices for scleral biomechanics are lacking [8]. Since both the cornea and sclera are derived from the mesoderm [9] and corneal deformation can partially reflect scleral mechanical properties [10], corneal biomechanical parameters are often utilized in research on assessing scleral biomechanics.

Some studies have utilized the Ocular Response Analyzer (ORA) to analyze the relationship between corneal biomechanics and myopia. This non-invasive device measures intraocular pressure (IOP) and corneal biomechanical properties. Studies have found that higher myopia is associated with lower corneal hysteresis (CH), a lower corneal resistance factor (CRF), and higher IOP [11,12]. Two meta-analyses suggested that low corneal biomechanics and high IOP may be contributing factors to high myopia [8,13]. However, some studies have reported that changes in CH and CRF were minimal with increasing refractive error, especially when compared to inter-subject variations or other potential influencing factors [14]. These studies often involved relatively small sample sizes. A recent UK Biobank study found that corneal biomechanics begin to decline at approximately −3.00 D, with exponential changes as myopia increases [6]. While various studies have explored the relationship between corneal biomechanics and myopia, some have suggested that lower corneal biomechanical properties may increase the risk of developing high myopia [13], whereas others have proposed that the progression of myopia, particularly to high levels, may lead to alterations in corneal biomechanics [6]. However, these correlation-based studies cannot address the fundamental question of causality—whether corneal biomechanics are the cause or the consequence of myopia [15]. This highlights the need for further investigation into the potential causal link between the two.

Mendelian randomization (MR) is a useful tool for identifying the causal effect of exposure on an outcome [16]. MR uses genetic variations as instrumental variables and relies on equally, randomly, and independently distributed genetic variants during meiosis, effectively avoiding the influence of confounding and reverse causation. Genome-wide association studies (GWAS) have identified thousands of genetic variations related to various complex diseases, pushing the widespread use of MR to an increasingly high stage [17].

This study aims to fill the gap in the current literature by leveraging a large dataset from the UK Biobank to investigate the relationship between myopia and corneal biomechanics. By employing MR analysis, we seek to elucidate the causal pathways that link myopia to changes in corneal biomechanical properties, thereby contributing to a deeper understanding of the etiology of myopia and informing the development of targeted prevention and treatment strategies.

## 2. Methods

### 2.1. Study Cohort

The UK Biobank (UKB) cohort includes over 500,000 participants, aged 37–73 at baseline (2006–2010), and it has detailed study protocols and test procedures available online “https://www.ukbiobank.ac.uk (accessed on 12 March 2024)”. Ethical approval for the UKB was granted by the Northwest Multi-Centre Research Ethics Committee (06/MRE08/65), and so no separate approval was required for this study. In total, 68,508 participants in the UKB underwent standardized ophthalmic assessments, including measurements from the Ocular Response Analyzer (ORA) (Reichert Corp., Philadelphia, PA, USA), which provides noninvasive assessments of IOP and corneal biomechanics. Participants without data on spherical equivalent (SE), CH, CRF, Goldmann-correlated IOP (IOPg), or corneal compensated IOP (IOPcc) were excluded. Additionally, outliers for CH, CRF, IOPg, and IOPcc—defined as values outside of Q1 − 1.5IQR or Q3 + 1.5IQR—were removed to account for potential measurement errors. To avoid surgical confounding, individuals with a history of ocular surgery were excluded. One eye per participant was randomly selected from the cleaned dataset, yielding a total of 18,522 eyes. Participants with hyperopia were then excluded based on SE, resulting in a final sample of 11,064 eyes. These participants were categorized into emmetropia and myopia groups based on their SE.

### 2.2. Statistical Analysis

Based on SE, the included eyes were classified into the following groups: emmetropia group (−0.5 < SE ≤ 0.5), mild myopia group (−3.0 < SE ≤ −0.5), moderate myopia group (−6.0 < SE ≤ −3.0), and high myopia group (SE ≤ −6.0). A normal Q-Q plot was used to assess the normality of the distribution of CH, CRF, IOPg, IOPcc, and age. Levene’s test was used to assess the homogeneity of the variances. Depending on the data distribution and variance homogeneity, one-way ANOVA or a Kruskal–Wallis test was employed to compare the differences among the different myopia severity groups. For post hoc pairwise comparisons, Tukey’s HSD test was used when the assumption of homogeneity of variances was met, while the Games–Howell test was used when it was violated. The above statistical analyses were performed using SPSS software (version 24.0, IBM Corp., Armonk, NY, USA). SE, CH, CRF, age, and IOPg were first standardized (we transformed the mean of the data to 0 and the standard deviation to 1), and then quantile regression was performed to examine the correlations between SE and CH and SE and CRF across the different quantiles (ranging from 0.02 to 0.98), with age and IOPg included as covariates (using Python version 3.13). Polynomial fitting was then performed on the original parameter values and quantile regression coefficients.

### 2.3. Mendelian Randomization (MR) Analysis Overview

**Figure 1:**
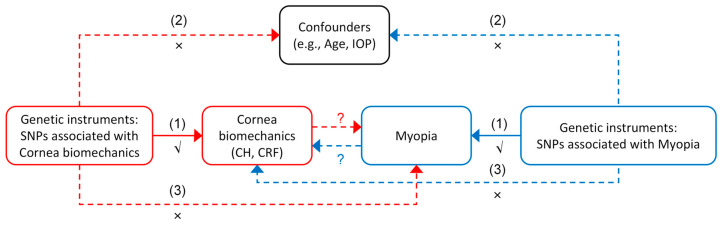
Overview of the bidirectional Mendelian randomization (MR) study design assessing the causal relationship between corneal biomechanics and myopia. The red and blue arrows represent the two directions of the MR analysis: from corneal biomechanics (CH, corneal hysteresis; CRF, corneal resistance factor) to myopia (red), and from myopia to corneal biomechanics (blue). The solid arrows labeled (1) indicate the primary causal pathways being tested. The dashed arrows labeled (2) and (3) represent the potential violations of the MR assumptions: (2) independence assumption—the genetic instruments should not be associated with confounders (e.g., age and intraocular pressure (IOP)); and (3) exclusion restriction assumption—the genetic instruments should influence the outcome only through the exposure, not via alternative pathways. Symbols: √ denotes that the relevance assumption is satisfied; × indicates a potential violation of assumptions; and ? indicates the direction of causality under investigation.

This study utilized GWAS data from the IEU Open GWAS project “https://gwas.mrcieu.ac.uk/ (accessed on 28 October 2024)” to explore the relationship between corneal biomechanics and myopia within the UKB cohort. This study was designed as a single-sample MR analysis, and an overview is illustrated in Figure 1. To ensure the validity of causal inference in the MR analyses, the genetic instrumental variables must have met three key assumptions: (1) relevance assumption: the instrumental variables must be strongly associated with the exposure; (2) independence assumption: the instrumental variables must not be associated with confounders; and (3) exclusion-restriction assumption: the instrumental variables must affect the outcome only through the exposure, without exerting significant direct effects or influencing the outcome through other confounding pathways. The MR analysis was conducted to investigate two key relationships: first, whether reduced corneal biomechanics increase susceptibility to myopia, and second, whether high myopia leads to reduced corneal biomechanics.

### 2.4. Instrumental Variables Selection

Single nucleotide polymorphisms (SNPs) are the most common genetic instrumental variables in the human genome. SNPs that were significantly associated with exposures (*p* < 5 × 10⁻^8^) were selected for further analysis. Linkage disequilibrium (LD) clumping was applied to these SNPs using a stringent threshold of R^2^ = 0.001 within a 10,000 kb window. To assess the potential for weak instrument bias, the F statistic was calculated and the SNPs with an F statistic of below 10 were excluded. If an SNP was not available in the outcome datasets, a proxy SNP with a minimum LD R^2^ of 0.8 was used as a substitute. For palindromic SNPs, strand alignment was attempted, and a minor allele frequency threshold of 0.3 was applied.

### 2.5. Data Analysis of the MR

The MR analysis was primarily conducted using the inverse variance weighted (IVW) method, which assumes no average pleiotropy. Heterogeneity was assessed using Cochran’s Q statistic, with a *p* value of < 0.05 indicating significant heterogeneity. In such cases, a multiplicative random-effects IVW model was applied to account for potential heterogeneity in effect estimates; otherwise, a fixed-effects IVW model was used. To enhance the robustness of the causal inference and minimize the impact of weak instrument bias and reverse causality—common challenges in a one-sample MR—we applied complementary MR methods and conducted additional sensitivity analyses, including: (1) MR-Egger regression, which can detect and adjust for directional pleiotropy by estimating an intercept that reflects the average pleiotropic effect across instruments, where a significant deviation in the intercept from zero (*p* value of < 0.05) indicates the presence of directional pleiotropy, suggesting that genetic variants may affect the outcome through pathways independent of the exposure; (2) the weighted median method, which provides a consistent and unbiased estimate of the causal effect even if up to 50% of the instruments are invalid as this method ranks the ratio estimates from all SNPs and uses the median as the causal effect estimate, making it less sensitive to outliers or pleiotropic instruments compared to IVW; and (3) a leave-one-out (LOO) analysis, which was used to assess the robustness of the causal estimate by sequentially excluding each SNP from the instrument set. This approach helps determine whether the observed association is driven by any single influential SNP and evaluates the overall stability of the MR results. Among these, IVW, MR-Egger, and weighted median are used to estimate the causal effect, whereas an LOO analysis is a sensitivity analysis designed to evaluate the influence of individual SNPs and does not provide an independent effect estimate.

Statistical analyses were conducted using the MR-Base platform, which integrates harmonized summary data from GWAS. The platform enables the implementation of MR analyses, including features such as effect allele harmonization and LD pruning to ensure the independence of genetic variants. All analyses were conducted on the MR-Base platform, following its terms and conditions to perform systematic causal inference [18].

Effect estimates were reported as β values with 95% confidence intervals (CIs) and converted to odds ratios (ORs) through exponential transformation, providing an intuitive interpretation of the results. All statistical tests were two-tailed, and α = 0.05 was considered as the significant level.

## 3. Results

Among the 11,064 participants, 5543 were female and 5521 were male, and the subjects were divided into four groups based on the severity of myopia, as follows: 4524 emmetropic, 3857 mild myopic, 1912 moderate myopic, and 771 high myopic eyes. Based on the results of the normal Q-Q plots, age, CH, CRF, IOPg, and IOPcc followed a normal distribution. A one-way ANOVA analysis revealed significant differences in the corneal biomechanical parameters and IOP values across the different severities of myopia (Table 1). CH was reduced in the high myopia group, and IOP increased with the progression of myopia. There was a significant age difference between the emmetropia and moderate myopia groups, while no statistically significant age differences were found among the other groups.

**Figure 2:**
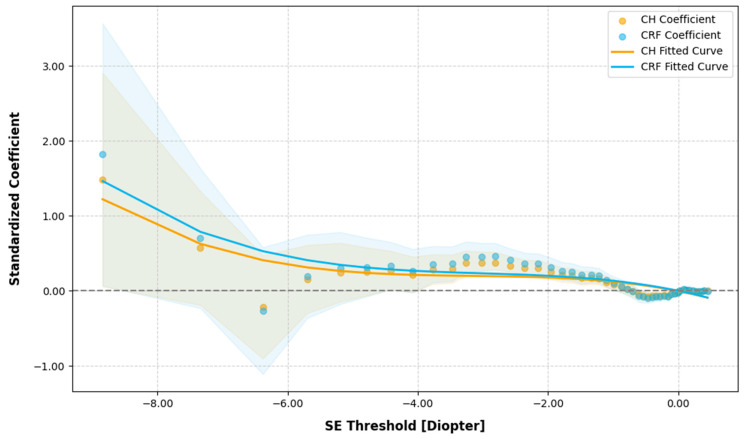
Quantile regression analysis of corneal hysteresis (CH) and corneal resistance factor (CRF) variations against spherical equivalent (SE) across the different SE quantiles. The grey dotted line represents the zero reference line for regression coefficients.

In the quantile regression analysis, in examining the effect of CH and CRF variations on SE across different SE quantiles, most standardized coefficients for CH and CRF were positive, indicating a significant positive correlation between CH, CRF, and SE (as shown in the supplementary Table S1). As CH and CRF decreased, SE decreased, reflecting a progression in the severity of myopia. The standardized coefficients for CH and CRF increased in magnitude as SE became more negative, corresponding to more severe myopia. For instance, when the SE was approximately −1.0 D, a one standard deviation increase in CH corresponded to a 0.080 D increase in SE, and at −3.0 D, the increase in SE was 0.367D, while at −8.842D, the increase was 1.480 D. Polynomial fitting of the quantile regression coefficients yielded the following equations: CH = −0.00526 SE^3^ + −0.05000 SE^2^ + −0.16970 SE + −0.00649 and CRF = −0.00550 SE^3^ + −0.05000 SE^2^ + −0.17817 SE + −0.00206 (as shown in Figure 2).

**Figure 3:**
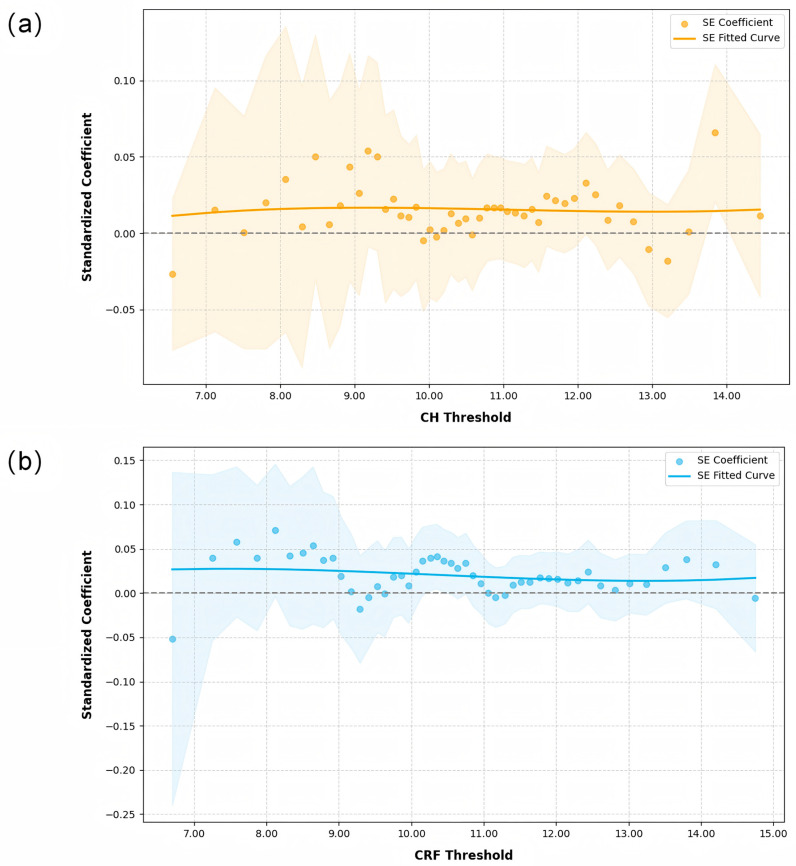
Quantile regression analysis of spherical equivalent (SE) variations against corneal hysteresis (CH) and corneal resistance factor (CRF) across the different CH and CRF quantiles: (**a**) CH, and (**b**) CRF. The grey dotted line represents the zero reference line for regression coefficients.

When analyzing the effect of SE variations on CH and CRF across the different CH and CRF quantiles, the results (Figure 3) showed a positive correlation between SE and CH/CRF throughout the entire range, but the effect was not significant (*p* > 0.05).

### 3.1. Effect of Cornea Biomechanics on Myopia

**Figure 4:**
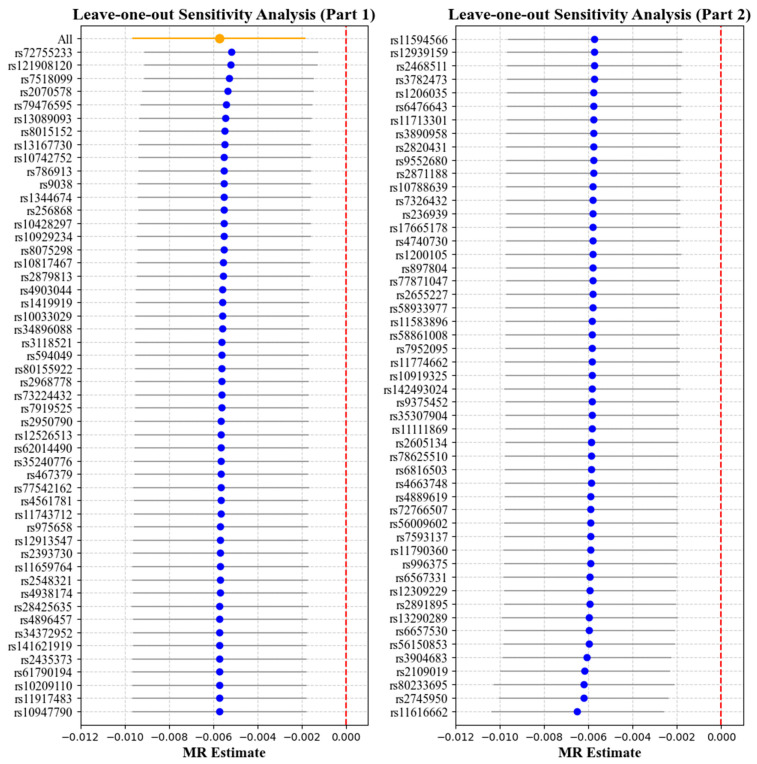
Leave-one-out analysis with corneal hysteresis (CH) as the exposure and myopia as the outcome. Each point represents the causal effect estimate (Mendelian randomization estimate) obtained by removing one single-nucleotide polymorphism (SNP) at a time. The *x*-axis shows the Mendelian randomization (MR) effect size and the *y*-axis lists the SNPs used as genetic instruments in the analysis. The orange point and line show the overall MR estimate and 95% CI using all SNPs; blue points represent leave-one-out estimates, with grey lines indicating their 95% CIs; the red dashed line marks the null effect (MR estimate = 0).

**Figure 5:**
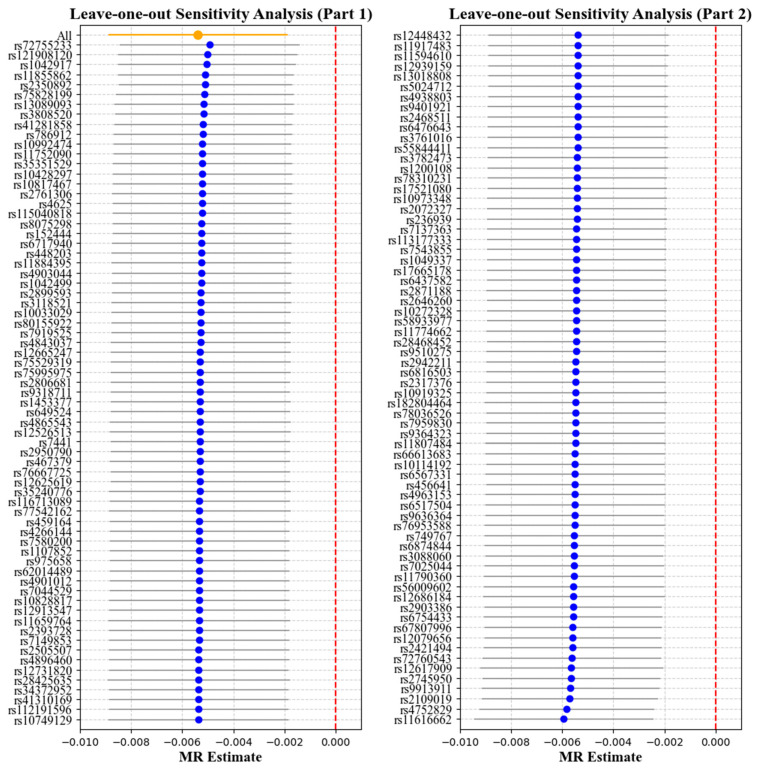
Leave-one-out analysis with corneal resistance factor (CRF) as the exposure and myopia as the outcome. Each point represents the causal effect estimate (Mendelian randomization estimate) obtained by removing one SNP at a time. The *x*-axis shows the Mendelian randomization (MR) effect size and the *y*-axis lists the SNPs used as genetic instruments in the analysis. The orange point and line show the overall MR estimate and 95% CI using all SNPs; blue points represent leave-one-out estimates, with grey lines indicating their 95% CIs; the red dashed line marks the null effect (MR estimate = 0).

Table 2 and Table 3 report the causal effect estimates obtained using different MR estimation methods, while Figure 4 and Figure 5 present the leave-one-out sensitivity analyses used to assess the robustness of these estimates. Heterogeneity was detected in the genetic variants associated with corneal biomechanics and myopia (CH: Cochran’s Q = 174, *p* < 0.001; CRF: Cochran’s Q = 265.9, *p* < 0.001). Therefore, the IVW method under multiplicative random effects was used to estimate the associations, demonstrating that genetically predicted lower CH and CRF values were associated with an increased risk of myopia (CH: OR = 0.9943, 95% CI = 0.9904–0.9982, *p* = 0.004; CRF: OR = 0.9946, 95% CI = 0.9912–0.9981, *p* = 0.002). The MR-Egger regression intercept term indicated no obvious directional pleiotropy existing among the SNPs in the two datasets, and the *p* values were both greater than 0.05. The weighted median and leave-one-out analyses both demonstrated the risk effect of cornea biomechanics on myopia and provided evidence of the stability of the results from the IVW method.

### 3.2. Effect of Myopia on Cornea Biomechanics

**Figure 6:**
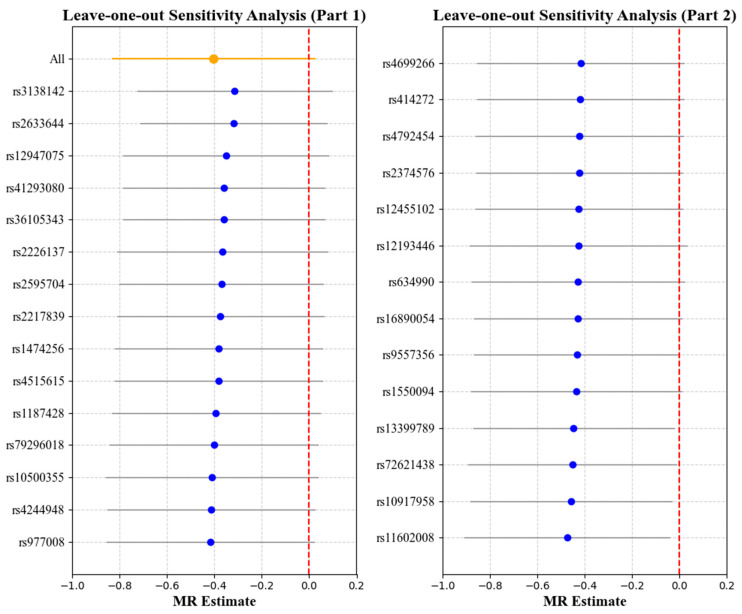
Leave-one-out analysis with myopia as the exposure and corneal hysteresis (CH) as the outcome. Each point represents the causal effect estimate (Mendelian randomization estimate) obtained by removing one SNP at a time. The *x*-axis shows the Mendelian randomization (MR) effect size and the *y*-axis lists the SNPs used as genetic instruments in the analysis. The orange point and line show the overall MR estimate and 95% CI using all SNPs; blue points represent leave-one-out estimates, with grey lines indicating their 95% CIs; the red dashed line marks the null effect (MR estimate = 0).

**Figure 7:**
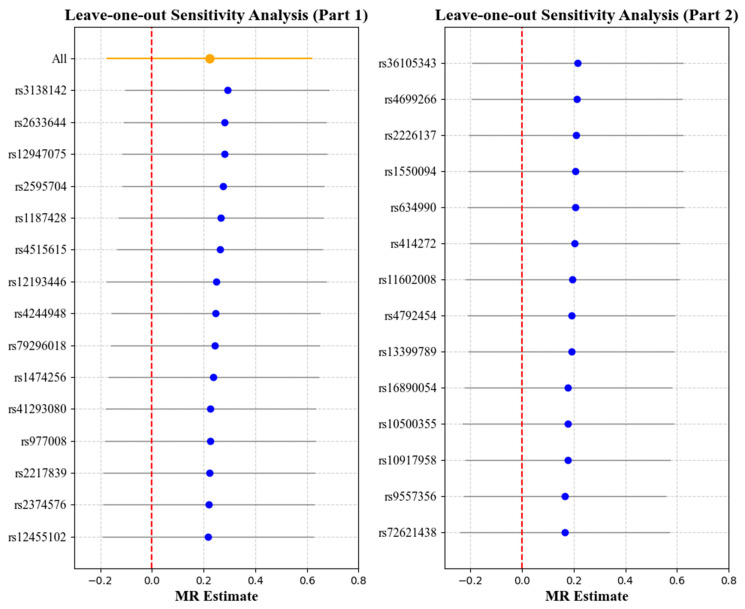
Leave-one-out analysis with myopia as the exposure and corneal resistance factor (CRF) as the outcome. Each point represents the causal effect estimate (Mendelian randomization estimate) obtained by removing one SNP at a time. The *x*-axis shows the Mendelian randomization (MR) effect size and the *y*-axis lists the SNPs used as genetic instruments in the analysis. The orange point and line show the overall MR estimate and 95% CI using all SNPs; blue points represent leave-one-out estimates, with grey lines indicating their 95% CIs; the red dashed line marks the null effect (MR estimate = 0).

Table 4 and Table 5 report the causal effect estimates obtained using the different MR estimation methods, while Figure 6 and Figure 7 present the leave-one-out sensitivity analyses used to assess the robustness of these estimates. No obvious heterogeneity was detected in the genetic variants associated with myopia and cornea biomechanics (CH: Cochran’s Q = 33.66, *p* = 0.2124; CRF: Cochran’s Q = 29.33, *p* = 0.3957). Therefore, the IVW method under fixed effects was applied to estimate the associations. The results indicated that non-myopia is a protective factor associated with higher CH values (OR = 0.6680, 95% CI = 0.4526–0.9860, *p* = 0.004), while, in combining the results from the weighted median and leave-one-out analysis, no causal relationship was observed when myopia was the exposure and CH was the outcome (*p* > 0.05) (Table 4, Figure 6). No causal relationship was observed between myopia and CRF (OR = 1.2488, 95% CI = 0.8485–1.8381, *p* = 0.2598) (Table 5, Figure 7).

## 4. Discussion

Understanding the relationship between corneal biomechanics and myopia is essential for exploring the mechanisms behind myopia progression and identifying potential biomarkers to assess the risk of high myopia. This study provides a comprehensive analysis of the bidirectional relationship between corneal biomechanics and myopia using multiple statistical approaches.

The study found that the high myopia group exhibited significantly lower corneal biomechanical values, with CH showing the most substantial reduction. Additionally, IOP increased in parallel with myopia severity. The quantile regression analysis demonstrated that lower corneal biomechanical values were associated with higher degrees of myopia, with the impact of corneal biomechanics becoming more pronounced as myopia severity increased. In contrast, when evaluating the reverse relationship—myopia’s influence on corneal biomechanics—the results indicated that SE had a minimal effect on CH and CRF across their full ranges.

These findings align with previous research that identified a correlation between reduced corneal biomechanics and increased myopia risk. Several observational studies have reported that lower corneal biomechanical properties are often associated with a greater severity of myopia, and SE has been positively correlated with CH and CRF in a linear regression model [11,14]. Furthermore, this pattern has also been observed in some large-scale meta-analyses, suggesting that corneal biomechanical properties may play a role in myopia progression [8,13]. However, a recent UK Biobank study using Ordinary Least Squares and Quantile Regression found that corneal biomechanics begin to deteriorate at approximately −3.00 D, with these changes increasing exponentially rather than linearly as myopia increases [6]. The Quantile Regression analysis in the current study similarly confirmed this pattern.

Although numerous studies have reported associations between corneal biomechanics and myopia, these findings have been based on observational data which establish correlation but not causation. Without causal inference methods, it remains unclear whether changes in corneal biomechanics contribute to myopia progression or if myopia leads to reduced corneal biomechanics. Traditional observational studies cannot confirm a bidirectional relationship due to limitations like confounding and reverse causation. Therefore, rigorous methods like MR are essential for determining the directional influence between corneal biomechanics and myopia.

This study utilized MR to assess causality in the relationship between corneal biomechanics and myopia. By using genetic variants as instrumental variables, MR reduces confounding and reverse causation, providing stronger evidence than observational studies. To ensure methodological rigor, multiple MR techniques—such as IVW, MR-Egger, and the weighted median approach—each addressing potential pleiotropy and bias, were applied to evaluate consistency and sensitivity. The bidirectional MR analysis confirmed that reduced corneal biomechanical properties increase myopia risk, while no causal effect was found when myopia was treated as the exposure and corneal biomechanics as the outcome. This suggests that reduced corneal biomechanics may contribute to myopia development rather than being a consequence of it. Our findings align with recent work by Wei et al. [19], reinforcing the genetic association between myopia and corneal biomechanics. Beyond the MR analysis, we applied quantile regression to assess severity-dependent variations and performed a leave-one-out analysis to refine the genetic instruments and address pleiotropy. These complementary approaches offered a detailed perspective on biomechanical changes in advanced myopia, supporting CH and CRF as potential biomarkers for high myopia risk assessment.

The observed association between corneal biomechanics and myopia risk may suggest that biomechanical changes in the eye represent fundamental alterations driving myopia progression. Given that both the cornea and sclera are composed of collagen fibers [20,21], corneal biomechanics may partially reflect the eye’s overall biomechanical changes [10,22]. A previous study found that lower CH and CRF values were significantly associated with longer axial length, suggesting that corneal biomechanics parameters are key indicators of both anterior and posterior ocular biometrics [15]. CRF reflects rigidity and resistance to deformation [23], and its decrease indicates compromised structural stability in myopic eyes, potentially due to reduced collagen fiber density in the corneal and scleral stroma. Previous animal studies have shown that high myopia is associated with decreased transforming growth factor-beta (TGF-β) [24], leading to biochemical changes in the sclera [25], such as reduced collagen synthesis and smaller collagen fiber diameters. CH indicates viscoelasticity [23], and a decline in CH reflects diminished recovery capacity under stress. In form-deprivation myopia models [26], the sclera has shown increased extensibility and permeability, leading to reduced fluid pressurization, collagen fiber relaxation, and weakened stability under dynamic loading.

The results also revealed that higher myopia was correlated with increased IOP, consistent with a majority of the previous studies that have reported a positive association between myopia severity and elevated IOP [11,13,27]. The mechanism underlying elevated intraocular pressure in myopic eyes remains unclear but may be due to increased wall stress following axial elongation, where reduced ocular rigidity and diminished resistance to external forces contribute to this elevation [28]. These changes suggest that in fluctuating IOP conditions, highly myopic eyes may be more prone to permanent deformation, affecting ocular shape and stability [29]. This also explains the nonlinear influence of biomechanics on myopia severity observed in UKB-based studies, which stems from the synergistic effects of reduced CH and increased IOP. In highly myopic eyes, a lower CH reflects diminished viscoelastic damping, amplifying the susceptibility to IOP-induced deformation. Additionally, an elevated IOP further exacerbates scleral and corneal creep [30], leading to accelerated axial elongation [31]. This dynamic feedback loop may explain why biomechanical changes in highly myopic eyes result in disproportionately greater myopia progression compared to lower myopic eyes. Based on foundational studies and this bidirectional analysis, corneal biomechanical changes appear to reflect broader pathological alterations in the eye rather than being a direct consequence of axial elongation in myopia. Therefore, eyes with lower corneal biomechanics have increased susceptibility to myopia, making the easily measurable corneal biomechanics a potential marker for high myopia risk.

Under this mechanism, enhancing the deformation resistance of ocular tissues to stabilize the structure of myopic eyes may help prevent further myopia progression. Clinically, scleral collagen cross-linking has been explored to enhance scleral strength and improve its biomechanical properties. This technique uses ultraviolet light and a photosensitizer to promote covalent bonding between collagen fibers, thereby slowing the progression of myopia [32].

Evaluating the relationship between corneal biomechanics and myopia not only aids in the risk assessment of myopia using biomechanical properties of the cornea as a substitute for the sclera but also helps evaluate treatments related to myopia correction and prevention, such as corneal refractive surgery [33] and orthokeratology [34]. For patients with high myopia, where substantial amounts of corneal tissue are removed in refractive surgery, analyzing corneal biomechanical properties is particularly essential [35]. To minimize the risk of postoperative corneal ectasia, patients considering refractive surgery should exercise caution when corneal biomechanics are low, myopia is high, and progression is ongoing. Additionally, in orthokeratology, the most commonly used approach for myopia control, corneal biomechanics influence the effectiveness of myopia control [36]. This is because the reshaping effect of orthokeratology depends on the corneal flattening, which is related to corneal biomechanics. By assessing corneal biomechanics, the effectiveness of orthokeratology in slowing myopia progression can be predicted [37,38]. Therefore, monitoring ocular biomechanics is beneficial not only for assessing myopia risk but also for evaluating the therapeutic effects and prognosis of orthokeratology.

There are several limitations that need to be mentioned. While MR provides a strong framework for causal inference, potential residual confounding cannot be entirely excluded. For example, some factors such as environmental exposures, lifestyle differences (e.g., screen time or outdoor activities), and comorbidities not included in the analysis may have influenced the observed associations. However, a series of thorough analyses were conducted to address potential horizontal pleiotropy and no significant pleiotropic effects were detected, supporting the validity of our instrumental variables. Additionally, the generalizability of the findings may be limited by the potential selection bias inherent in the UKB cohort. Furthermore, the relatively high mean age of the study population may have introduced age-related changes in corneal biomechanics, potentially influencing the observed associations. Although age was included as a covariate in the regression models, this age structure limits the extrapolation of findings to younger populations. These limitations highlight the need for further validation in diverse populations and datasets. Additionally, using corneal biomechanics (CH and CRF) as proxies for scleral biomechanics, which are more directly related to myopia progression, may limit the interpretation of the findings, as corneal metrics may not fully capture scleral biomechanical properties. Future research could focus on developing direct scleral biomechanics measurement methods [29,39], offering a clearer understanding of how scleral changes influence myopia progression. Such advancements could enhance diagnostic and prognostic tools, enabling early intervention and more targeted management for individuals at risk of high myopia.

## 5. Conclusions

This study provides valuable insights into the relationship between corneal biomechanics and myopia and is supported by a large database analysis. The findings indicate that lower CH and CRF values are associated with an increased risk of myopia, with the MR analysis suggesting a causal role for reduced corneal biomechanics that contributes to myopia, while myopia does not directly affect biomechanics. Highlighting corneal biomechanics as a potential biomarker for high myopia risk, the findings offer implications for early diagnosis and preventive strategies. Future studies should explore direct assessments of scleral biomechanics for a more comprehensive understanding of ocular biomechanics in myopia.

## Figures and Tables

**Table 1:** Comparison of corneal biomechanics, intraocular pressure, and age across the groups with differing severities of myopia.

Parameter	Group	Mean ± SD	*p* Value
Corneal hysteresis (CH)	Emmetropia	10.48 ± 1.88 a	<0.001
	Mild myopia	10.52 ± 1.89 a	
	Moderate myopia	10.60 ± 1.86 a	
	High myopia	10.26 ± 1.95 b	
Corneal resistance factor (CRF)	Emmetropia	10.50 ± 1.94 a	<0.001
	Mild myopia	10.62 ± 1.98 b	
	Moderate myopia	10.85 ± 1.97 c	
	High myopia	10.64 ± 2.03 ab	
Goldmann-correlated intraocular pressure (IOPg)	Emmetropia	15.58 ± 3.50 a	<0.001
	Mild myopia	15.85 ± 3.54 b	
	Moderate myopia	16.40 ± 3.53 c	
	High myopia	16.65 ± 3.50 c	
Corneal compensated intraocular pressure (IOPcc)	Emmetropia	15.97 ± 3.57 a	<0.001
	Mild myopia	16.16 ± 3.57 a	
	Moderate myopia	16.54 ± 3.53 b	
	High myopia	17.13 ± 3.54 c	
Age	Emmetropia	59.71 ± 7.90 a	0.015
	Mild myopia	60.03 ± 7.54 ab	
	Moderate myopia	60.35 ± 7.28 b	
	High myopia	59.94 ± 7.36 ab	

The different superscript letters (a, b, and c) indicate significant group differences based on post hoc comparisons (*p* < 0.05). For CH, CRF, IOPg, and IOPcc (with equal variances), Tukey’s HSD test was used for pairwise comparisons. For age, the Levene’s test showed unequal variances (*p* < 0.001). Thus, Welch’s ANOVA was applied to test for group differences, followed by Games–Howell post hoc tests for pairwise comparisons.

**Table 2:** Different Mendelian randomization (MR) methods for corneal hysteresis (CH) as the exposure and myopia as the outcome.

MR Method	nSNP	β	Standard Error	*p* Value
Inverse variance weighted (multiplicative random effects)	102	−0.005733	0.001981	0.0038
Weighted median	102	−0.005735	0.002597	0.0272

**Table 3:** Different Mendelian randomization (MR) methods for corneal resistance factor (CRF) as the exposure and myopia as the outcome.

MR Method	nSNP	β	Standard Error	*p* Value
Inverse variance weighted (multiplicative random effects)	137	−0.005383	0.001774	0.0024
Weighted median	137	−0.006051	0.002127	0.0044

**Table 4:** Different Mendelian randomization (MR) methods for myopia as the exposure and corneal hysteresis (CH) as the outcome.

MR Method	nSNP	β	Standard Error	*p* Value
Inverse variance weighted (fixed effects)	29	−0.4034	0.1986	0.04221
Weighted median	29	−0.1375	0.2778	0.6207

**Table 5:** Different Mendelian randomization (MR) methods for myopia as the exposure and corneal resistance factor (CRF) as the outcome.

MR Method	nSNP	β	Standard Error	*p* Value
Inverse variance weighted (fixed effects)	29	0.2222	0.1972	0.2598
Weighted median	29	0.4306	0.2696	0.1101

## Data Availability

This research was conducted using data from the UK Biobank. Due to UK Biobank’s access control policies, data directly supporting the results of this work are only accessible by the immediate research team members. However, bona fide researchers can apply for access to the data through the UK Biobank at https://www.ukbiobank.ac.uk/enable-your-research/apply-for-access (accessed on 12 March 2024).

## Supplementary Materials

The following supporting information can be downloaded at: https://www.mdpi.com/article/10.3390/bioengineering12040412/s1, Table S1: Quantile regression between spherical equivalent (SE) and corneal hysteresis (CH), and corneal resistance factor (CRF) across different quantiles.

## Abbreviations

UKB	UK Biobank
ORA	Ocular Response Analyzer
CH	Corneal hysteresis
CRF	Corneal resistance factor
IOP	Intraocular pressure
IOPcc	Corneal-compensated intraocular pressure
IOPg	Goldmann-correlated intraocular pressure
SE	Spherical equivalent
MR	Mendelian randomization
OR	Odds ratios
GWAS	Genome-wide association studies
SNPs	Single-nucleotide polymorphisms
LD	Linkage disequilibrium
IVW	Inverse variance weighted
LOO	Leave-one-out
CI	Confidence interval

## References

[1] Holden B.A., Fricke T.R., Wilson D.A., Jong M., Naidoo K.S., Sankaridurg P., Wong T.Y., Naduvilath T.J., Resnikoff S. (2016). Global Prevalence of Myopia and High Myopia and Temporal Trends from 2000 through 2050. Ophthalmology.

[2] Morgan I.G., Wu P.C., Ostrin L.A., Tideman J.W.L., Yam J.C., Lan W., Baraas R.C., He X., Sankaridurg P., Saw S.M. (2021). IMI Risk Factors for Myopia. Invest. Ophthalmol. Vis. Sci..

[3] Naidoo K.S., Fricke T.R., Frick K.D., Jong M., Naduvilath T.J., Resnikoff S., Sankaridurg P. (2019). Potential Lost Productivity Resulting from the Global Burden of Myopia: Systematic Review, Meta-analysis, and Modeling. Ophthalmology.

[4] Curtin B.J., Teng C.C. (1958). Scleral changes in pathological myopia. Trans. Am. Acad. Ophthalmol. Otolaryngol..

[5] Curtin B.J., Iwamoto T., Renaldo D.P. (1979). Normal and staphylomatous sclera of high myopia. An electron microscopic study. Arch. Ophthalmol..

[6] Yii F., Strang N., Bernabeu M.O., Dhillon B., MacGillivray T. (2024). Corneal biomechanics are not exclusively compromised in high myopia. Ophthalmic Physiol. Opt..

[7] Norman R.E., Flanagan J.G., Rausch S.M., Sigal I.A., Tertinegg I., Eilaghi A., Portnoy S., Sled J.G., Ethier C.R. (2010). Dimensions of the human sclera: Thickness measurement and regional changes with axial length. Exp. Eye Res..

[8] Liu M.X., Zhu K.Y., Li D.L., Dong X.X., Liang G., Grzybowski A., Pan C.W. (2024). Corneal Biomechanical Characteristics in Myopes and Emmetropes Measured by Corvis ST: A Meta-Analysis. Am. J. Ophthalmol..

[9] McBrien N.A., Gentle A. (2003). Role of the sclera in the development and pathological complications of myopia. Prog. Retin. Eye Res..

[10] Nguyen B.A., Roberts C.J., Reilly M.A. (2018). Biomechanical Impact of the Sclera on Corneal Deformation Response to an Air-Puff: A Finite-Element Study. Front. Bioeng. Biotechnol..

[11] Jiang Z., Shen M., Mao G., Chen D., Wang J., Qu J., Lu F. (2011). Association between corneal biomechanical properties and myopia in Chinese subjects. Eye.

[12] Du Y., Zhang Y., Zhang Y., Li T., Wang J., Du Z. (2023). Analysis of potential impact factors of corneal biomechanics in myopia. BMC Ophthalmol..

[13] Wu W., Dou R., Wang Y. (2019). Comparison of Corneal Biomechanics Between Low and High Myopic Eyes-A Meta-analysis. Am. J. Ophthalmol..

[14] Plakitsi A., O’Donnell C., Miranda M.A., Charman W.N., Radhakrishnan H. (2011). Corneal biomechanical properties measured with the Ocular Response Analyser in a myopic population. Ophthalmic Physiol. Opt..

[15] Chang P.Y., Chang S.W., Wang J.Y. (2010). Assessment of corneal biomechanical properties and intraocular pressure with the Ocular Response Analyzer in childhood myopia. Br. J. Ophthalmol..

[16] Smith G.D., Ebrahim S. (2003). ‘Mendelian randomization’: Can genetic epidemiology contribute to understanding environmental determinants of disease?. Int. J. Epidemiol..

[17] Porcu E., Rueger S., Lepik K., Santoni F.A., Reymond A., Kutalik Z., eQTLGen Consortium, BIOS Consortium (2019). Mendelian randomization integrating GWAS and eQTL data reveals genetic determinants of complex and clinical traits. Nat. Commun..

[18] Hemani G., Zheng J., Elsworth B., Wade K.H., Haberland V., Baird D., Laurin C., Burgess S., Bowden J., Langdon R. (2018). The MR-Base platform supports systematic causal inference across the human phenome. eLife.

[19] Wei P., Han G., Su Q., Jia L., Xue C., Wang Y. (2025). Corneal biomechanics as a causal factor in myopia and astigmatism: Evidence from Mendelian randomization. Ophthalmol. Sci..

[20] Boote C., Sigal I.A., Grytz R., Hua Y., Nguyen T.D., Girard M.J.A. (2020). Scleral structure and biomechanics. Prog. Retin. Eye Res..

[21] Meek K.M., Fullwood N.J. (2001). Corneal and scleral collagens--a microscopist’s perspective. Micron.

[22] Bronte-Ciriza D., Birkenfeld J.S., de la Hoz A., Curatolo A., Germann J.A., Villegas L., Varea A., Martinez-Enriquez E., Marcos S. (2021). Estimation of scleral mechanical properties from air-puff optical coherence tomography. Biomed. Opt. Express.

[23] Elsheikh A., Joda A., Abass A., Garway-Heath D. (2015). Assessment of the Ocular Response Analyzer as an Instrument for Measurement of Intraocular Pressure and Corneal Biomechanics. Curr. Eye Res..

[24] Matsumura S., Kuo A.N., Saw S.M. (2019). An Update of Eye Shape and Myopia. Eye Contact Lens.

[25] Hoerig C., McFadden S., Hoang Q.V., Mamou J. (2022). Biomechanical changes in myopic sclera correlate with underlying changes in microstructure. Exp. Eye Res..

[26] Brown D.M., Kowalski M.A., Paulus Q.M., Yu J., Kumar P., Kane M.A., Patel J.M., Ethier C.R., Pardue M.T. (2022). Altered Structure and Function of Murine Sclera in Form-Deprivation Myopia. Invest. Ophthalmol. Vis. Sci..

[27] Chong R.S., Li H., Cheong A.J.Y., Fan Q., Koh V., Raghavan L., Nongpiur M.E., Cheng C.Y. (2023). Mendelian Randomization Implicates Bidirectional Association between Myopia and Primary Open-Angle Glaucoma or Intraocular Pressure. Ophthalmology.

[28] Schmid KL L.R. (2003). Edwards MH, Lew JK, The expandability of the eye in childhood myopia. Curr. Eye Res..

[29] Luo J., Zhang Y., Ai S., Shi G., Han X., Wang Y., Zhao Y., Yang H., Li Y., He X. (2024). Two-dimensional elastic distribution imaging of the sclera using acoustic radiation force optical coherence elastography. J. Biophotonics.

[30] Nguyen T.D., Jones R.E., Boyce B.L. (2008). A nonlinear anisotropic viscoelastic model for the tensile behavior of the corneal stroma. J. Biomech. Eng..

[31] Song D., Lim S., Park J., Demer J.L. (2023). Linear viscoelasticity of human sclera and posterior ocular tissues during tensile creep. J. Biomech..

[32] Yasir Z.H., Sharma R., Zakir S.M. (2024). Scleral collagen cross linkage in progressive myopia. Indian. J. Ophthalmol..

[33] Pniakowska Z., Jurowski P., Wierzbowska J. (2022). Clinical Evaluation of Corneal Biomechanics following Laser Refractive Surgery in Myopic Eyes: A Review of the Literature. J. Clin. Med..

[34] Zhang P., Wu J., Jiang J., Zhang X., Ran Z., Jiang F., Zheng X., Wang J., Elsheikh A., Bao F. (2024). Evaluation of changes in corneal biomechanics after orthokeratology using Corvis ST. Cont. Lens Anterior Eye.

[35] Dackowski E.K., Lopath P.D., Chuck R.S. (2020). Preoperative, intraoperative, and postoperative assessment of corneal biomechanics in refractive surgery. Curr. Opin. Ophthalmol..

[36] Lam A.K.C., Hon Y., Leung S.Y.Y., Shu-Ho L., Chong J., Lam D.C.C. (2019). Association between long-term orthokeratology responses and corneal biomechanics. Sci. Rep..

[37] Xiang K., Chen J., Zhao W., Zhu Z., Ding L., Bulloch G., Du L., Xu X., Zhu M., He X. (2023). Changes of corneal biomechanics in children using orthokeratology and their roles in predicting axial length progression-A prospective 2-year study. Acta Ophthalmol..

[38] Li X., Xu J., Hong J., Yao J. (2022). The relationship between corneal biomechanical parameters and treatment outcomes of orthokeratology lenses. BMC Ophthalmol..

[39] Mekonnen T., Zevallos-Delgado C., Singh M., Aglyamov S.R., Larin K.V. (2023). Multifocal acoustic radiation force-based reverberant optical coherence elastography for evaluation of ocular globe biomechanical properties. J. Biomed. Opt..

