# Supplementary material for: Causal Links Between Corneal Biomechanics and Myopia: Evidence from Bidirectional Mendelian Randomization in the UK Biobank
Source: Bioengineering (Basel). 2025 Apr 13;12(4):412. doi: 10.3390/bioengineering12040412 (PMC12024697; doi:10.3390/bioengineering12040412)
Supplement: Supplementary file 1 [file bioengineering-12-00412-s001.zip › bioengineering-3540343-supplementary.pdf]

Table S1 Quantile regression between spherical equivalent (SE) and corneal hysteresis (CH), and corneal resistance factor (CRF) across different quantiles

Quantile	SE	CH			CRF		
	Threshold	Coefficient	Std Error	P value	Coefficient	Std Error	P value
0.02	-8.842	1.480	0.728	0.043	1.817	0.893	0.043
0.04	-7.340	0.568	0.387	0.143	0.697	0.475	0.143
0.06	-6.380	-0.216	0.351	0.538	-0.265	0.431	0.539
0.08	-5.700	0.154	0.231	0.504	0.190	0.284	0.504
0.10	-5.190	0.245	0.200	0.221	0.301	0.245	0.220
0.12	-4.780	0.256	0.162	0.116	0.314	0.199	0.116
0.14	-4.420	0.271	0.132	0.040	0.332	0.162	0.041
0.16	-4.080	0.214	0.121	0.077	0.263	0.149	0.077
0.18	-3.770	0.285	0.101	0.005	0.350	0.124	0.005
0.20	-3.474	0.291	0.096	0.002	0.358	0.118	0.002
0.22	-3.260	0.368	0.084	<0.001	0.452	0.104	<0.001
0.24	-3.020	0.367	0.077	<0.001	0.450	0.095	<0.001
0.26	-2.810	0.372	0.074	<0.001	0.457	0.090	<0.001
0.28	-2.580	0.333	0.064	<0.001	0.408	0.079	<0.001
0.30	-2.360	0.298	0.057	<0.001	0.365	0.071	<0.001
0.32	-2.150	0.297	0.054	<0.001	0.364	0.067	<0.001
0.34	-1.956	0.255	0.053	<0.001	0.313	0.065	<0.001
0.36	-1.780	0.213	0.050	<0.001	0.261	0.061	<0.001
0.38	-1.630	0.203	0.048	<0.001	0.248	0.059	<0.001
0.40	-1.490	0.170	0.048	<0.001	0.208	0.059	<0.001
0.42	-1.340	0.173	0.044	<0.001	0.211	0.054	<0.001
0.44	-1.220	0.163	0.041	<0.001	0.199	0.051	<0.001
0.46	-1.100	0.116	0.039	0.003	0.142	0.048	0.003
0.48	-0.990	0.080	0.037	0.031	0.098	0.045	0.031
0.50	-0.870	0.049	0.037	0.180	0.060	0.045	0.180
0.52	-0.780	0.020	0.037	0.592	0.024	0.046	0.592
0.54	-0.700	-0.009	0.037	0.818	-0.010	0.045	0.818
0.56	-0.610	-0.053	0.035	0.129	-0.066	0.043	0.128
0.58	-0.540	-0.067	0.032	0.039	-0.082	0.040	0.039
0.60	-0.470	-0.076	0.029	0.008	-0.094	0.036	0.008
0.62	-0.400	-0.073	0.026	0.006	-0.088	0.032	0.006
0.64	-0.340	-0.064	0.024	0.008	-0.078	0.030	0.008
0.66	-0.280	-0.068	0.022	0.002	-0.084	0.027	0.002
0.68	-0.220	-0.060	0.020	0.003	-0.073	0.024	0.003
0.70	-0.160	-0.065	0.018	<0.001	-0.080	0.022	<0.001
0.72	-0.110	-0.042	0.016	0.009	-0.052	0.020	0.009
0.74	-0.060	-0.036	0.014	0.013	-0.043	0.018	0.014
0.76	-0.010	-0.024	0.013	0.061	-0.029	0.016	0.061
0.78	0.000	-0.004	0.011	0.696	-0.005	0.014	0.696
0.80	0.030	0.001	0.010	0.909	0.001	0.013	0.909

0.82	0.080	0.015	0.009	0.114	0.018	0.012	0.115
0.84	0.120	0.007	0.009	0.407	0.009	0.011	0.408
0.86	0.170	0.006	0.008	0.478	0.007	0.010	0.479
0.88	0.220	0.000	0.008	1.000	0.000	0.010	1.000
0.90	0.270	-0.007	0.007	0.300	-0.009	0.009	0.298
0.92	0.310	-0.006	0.007	0.364	-0.007	0.008	0.364
0.94	0.360	-0.004	0.006	0.524	-0.005	0.008	0.524
0.96	0.400	0.001	0.006	0.833	0.002	0.008	0.833
0.98	0.450	-0.003	0.005	0.526	-0.004	0.006	0.538

Quantile refers to the specific quantile level used in quantile regression. Threshold indicates the corresponding SE value at that quantile. Coefficient represents the estimated effect size of CH or CRF on SE at each quantile. Std. Error is the standard error of the coefficient estimate. P value assesses the statistical significance of the association.
